# A Comparative Evaluation of the New Genexpert MTB/RIF Ultra and other Rapid Diagnostic Assays for Detecting Tuberculosis in Pulmonary and Extra Pulmonary Specimens

**DOI:** 10.1038/s41598-019-53086-5

**Published:** 2019-11-12

**Authors:** John Osei Sekyere, Nontobeko Maphalala, Lesibana A. Malinga, Nontombi M. Mbelle, Nontuthuko E. Maningi

**Affiliations:** 1 Department of Medical Microbiology, University of Pretoria, 0084 Pretoria, South Africa; 2 National Health Laboratory Services, Tshwane Academic Division, 0084 Pretoria, South Africa; 3 South African Medical Research Council, TB Platform Unit, 0084 Pretoria, South Africa; 4 Department of Internal Medicine, University of Pretoria, 0084 Pretoria, South Africa

**Keywords:** Bacteriology, Clinical microbiology

## Abstract

Studies evaluating the new GeneXpert Ultra with other rapid diagnostic assays are limited, particularly in different geographical settings. The performance of the GeneXpert Ultra, the GeneXpert G4, the Line probe assays (LPA) and auramine smear microscopy in detecting TB in pulmonary and extra-pulmonary samples were thus evaluated. Remnants (n = 205 samples) of pulmonary (n = 125 samples) and extra-pulmonary (n = 80 samples) specimens from TB suspects were prospectively collected. Each sample was divided for diagnosis using microscopy, GeneXpert MTB/RIF assays, and LPA; these were all comparatively evaluated, using the MGIT 960 culture as a gold standard. The sensitivity and specificity of microscopy, Xpert Ultra, Xpert G4 and MTBDR*plus* (ver 2) in pulmonary samples were respectively: 82.00% and 90.28%; 88.00% and 58.57%; 79.59% and 90.28%; 80.00% and 11.11%. For extra-pulmonary specimen, the sensitivity and specificity were respectively: 53.85% and 98.51%; 69.23% and 49.25%; 50.00% and 97.01%; 69.23% and 25.37%. The new and improved GeneXpert Ultra assay was more sensitive than GeneXpert G4 and LPA in both pulmonary and extra pulmonary samples, albeit with lower specificity than the GeneXpert G4. The auramine and LPA tests were also highly sensitive, although the LPA was less specific.

## Introduction

Although curable, tuberculosis (TB) remains the deadliest infectious disease caused by a single aetiological agent, *Mycobacterium tuberculosis* (*M. tb*), among all infectious diseases. In 2017, 1.5 million people died of TB globally. In South Africa, HIV-negative TB deaths were estimated to be ~23000 (17000–29000) while HIV-positive mortalities totalled 101 000 (67 000-142 000) in 2017^1,2^. The WHO has reported a decline in TB incidence and mortality rates, and this has been due to the introduction of rapid diagnostic assays and effective TB treatment. Despite the global decline in new infections, the emergence of multidrug-resistant TB (MDR-TB) is worsening the burden of TB. In 2017, 558 000 people had MDR-TB, an increase from 490 000 people in 2016 while in South Africa, a decline from 19 000 cases in 2016 to 15 000 cases in 2017 was reported^1^. In South Africa, this decline was due to major efforts by the government to manage the problem, including the rolling out of GeneXpert machines throughout the country for quick diagnosis, screening high-risk groups (HIV infected individuals) to prevent transmission, treating latent TB, and using quality *M. tb* drugs for treatment^3^.

In 2010, the WHO recommended the use of the GeneXpert MTB/RIF G4 (hereafter referred to as Xpert G4), which is an automated cartridge-based molecular test, as a primary test to increase TB detection and improve diagnosis of rifampicin (RIF) resistance in pulmonary and extra-pulmonary TB (EPTB) specimens^4^. However, the Xpert G4 assay’s sensitivity for TB detection is inadequate when few bacilli are present in the specimens, particularly in vulnerable groups such as HIV-infected patients, children and in extra-pulmonary TB samples^5–7^. Moreover, in terms of RIF resistance detection, the Xpert MTB/RIF G4 cartridge assay gives false-positive results for strains that carry phenotypically silent mutations (synonymous mutations), or for paucibacillary specimens^8,9^. This has led to the limited usefulness of the Xpert MTB/RIF G4 cartridge assay in diagnosing TB and RIF resistance.

A new GeneXpert MTB/RIF Ultra (hereafter referred as Xpert Ultra) assay has been developed to overcome the limitations of the old Xpert MTB/RIF G4 assay with improved sensitivity in the detection of TB and RIF resistance. The Xpert Ultra assay is a rapid assay that uses an improved assay chemistry and cartridge design. It incorporates two different multicopy amplification targets viz., IS6110 and IS1081, and the RIF resistance-determining region (RRDR) of the *rpo*B gene^10,11^. These changes resulted in a ten-fold improvement in the lower limit of TB detection. Additionally, analytical laboratory data also showed an improved differentiation of certain silent mutations, improved detection of RIF resistance in mixed infections and reduced false-positive results in detecting RIF resistance in paucibacillary specimens.

Currently, only one multicentre study has been conducted to evaluate the performance of the new Xpert Ultra assay^10^. In that study, adults presenting with pulmonary TB symptoms at primary healthcare centres and hospitals in eight countries i.e., South Africa, Uganda, Kenya, India, China, Georgia, Belarus, and Brazil, were recruited. Reported findings showed that the sensitivity of Xpert Ultra was superior to that of Xpert G4 in patients with paucibacillary disease and in patients with HIV. However, an increase in sensitivity led to a decrease in specificity^10^. Thus, more studies are needed to assess the performance of the new Xpert MTB/RIF Ultra assay on pulmonary and extra-pulmonary samples in different geographical settings.

Besides the Xpert G4, the Xpert Ultra, the auramine smear microscopy and Hain’s line probe assay (LPA), which are also WHO-approved diagnostic tests for TB detection^12^, were compared using the BACTEC MGIT 960 culture assay as a gold standard in all cases. To our knowledge, no evaluation study has been undertaken to compare the Xpert Ultra to the smear microscopy and LPA, making this the first. This study therefore aims to provide additional and informative data on the diagnostic efficiencies of these assays to inform TB diagnostic choices, particularly in less-resourced clinical microbiology laboratories.

## Materials and Methods

### Study design and setting

**Figure 1:**
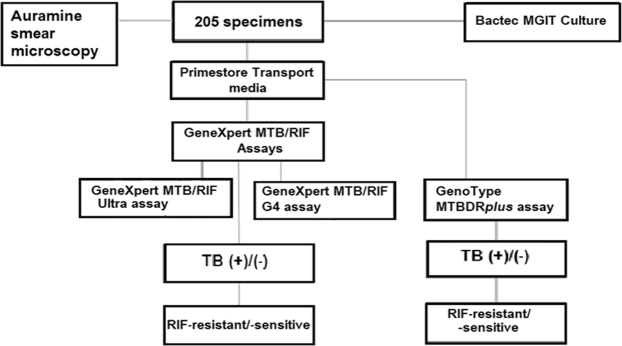
Flow diagram of methodologies used in processing samples and evaluating the diagnostic efficacies of smear microscopy, Xpert Ultra, Xpert G4 and LPA tests in detecting Mycobacterium tuberculosis from pulmonary and extra-pulmonary samples.

This study prospectively evaluated and compared the TB detection capabilities of the new GeneXpert MTB/RIF Ultra assay (Cepheid Inc, USA), the old GeneXpert MTB/RIF G4 (Cepheid Inc, USA), the GenoType MTBDR*plus* assay (Hain Lifescience, Nehren, Germany) and the auramine smear microscopy using BACTEC MGIT 960 (BD, Sparks MD, USA) culture as a gold standard (Fig. 1).

Remnants of pulmonary (bronchial alveolar lavage [BAL], trachea, trachea aspirate, and sputum)(n = 125) and extra-pulmonary (urine, superficial swabs, gastric aspirates, abscess, tissue, cerebrospinal fluid [CSF], and fluid aspirates [from non-pulmonary but unspecified/unknown anatomical site])(n = 80) clinical specimens were collected from the National Health Laboratory Services (NHLS), Tshwane Academic Division (NHLS/TAD) in Pretoria from patients who were suspected to be infected with TB and had come for their first TB screening. The volume of each specimen differed as remnant samples were used; hence, 800 µL of each specimen was used for the molecular assays. All remnant samples from persons suspected of having TB were included while those from doubtful sources, on treatment or had contaminations were discarded.

### Sample processing

The 205 remnant samples were equally divided into three aliquots (volumes): one was processed by decontamination using 1% NALC-NaOH method for culture into MGIT 7H9 (BD, Sparks MD, USA); the second was used for the auramine smear microscopy; and the third sample (800 µL per sample) was transferred into 1.8 mL PrimeStore (PS) molecular transport media (MTM) tubes (Longhorn Vaccines and Diagnostics LLC, USA) for molecular assays (Xpert and MTBDR*plus*) (Fig. 1). Each 1.8 mL PS-MTM tube contained 1 mL of PS-MTM. All samples were vortexed using the Vortex Genie 2 mixer (Scientific Industries Inc, USA) for 5–10 seconds. A volume of 800 µL of specimen was aliquoted into the correctly labelled 1.8 mL PS-MTM tube, mixed by pipetting up and down thrice and the tip was discarded after use. Finally, the PrimeStore tubes were vortexed with the specimen using Vortex Genie 2 mixer (Scientific Industries Inc, USA) and stored at room temperature for later use. The specimens stored in PrimeStore were divided into three: one specimen (200 µL) was used for DNA extraction for the LPA assay and the other two were used for the Xpert Ultra assay (500 µL) and for the old Xpert G4 assay (500 µL) according to manufacturer’s instructions^13,14^.

### Auramine smear microscopy

The second portion of the 205 specimens were subjected to the auramine smear microscopy by fixing the specimens unto slides, applying rhodamine auramine to the fixed slide for 5 minutes, and then rinsing it, applying the decolorizing agent, acid-alcohol, and then rinsing it, applying potassium permanganate for 2 minutes and then rinsing, drying and observing the slide with a fluorescent microscope as already described (Wanger *et al*., 2017).

### GeneXpert MTB/RIF assays

The GeneXpert assays (Ultra and G4) were conducted on all 205 (125 pulmonary and 80 extra-pulmonary) samples. A 1.0 mL of GeneXpert MTB/RIF assay sample reagent (Cepheid Inc, USA) was added to 500 µL of each specimen (already mixed with the PS-MTM) using a sterile pipette (Tang *et al*., 2017).

### DNA extraction

DNA was extracted from all 205 (125 pulmonary and 80 extra-pulmonary) specimens using PrimeXtract extraction kits (Long Horn Vaccines and Diagnostics, USA). A volume of 200 μL of 100% ethanol, 200 μL lysis buffer and 200 μL specimen (already mixed with the PS-MTM) were transferred into a 1.5 mL micro-centrifuge tube followed by thorough mixing and subsequent centrifugation. The entire supernatant was then pipetted into a micro extraction column, where it was centrifuged for 1 min at 13,000x g, and the flow-through material was discarded. A wash buffer (200 μL) was pipetted into the extraction column and centrifuged for 1 min at 13 000x g, followed by further addition of wash buffer (200 μL) to the extraction column, and subsequent centrifuging as described above, discarding the flow through material. The total *M. tb* DNA was eluted by centrifuging for 1 min at maximum speed using 50 μL of elution solution preheated at 75 °C. The DNA was stored at −20 °C for future use.

The GenoType MTBDR*plus* LPA test was conducted according to the manufacturer’s instructions. Briefly, amplification reactions were undertaken using a 35 µL primer nucleotide mix, 10 µL polymerase mix (Hain Lifescience, Germany), and 5 µL of genomic DNA. PCR and hybridization were performed and interpreted as per the manufacturer’s instructions using the GTBlot and GenoScan machines (Hain Lifescience, Germany).

### Sensitivity, specificity, and negative and positive predictive values calculations

Sensitivity, specificity, negative-predictive values (NPV) and positive-predictive values (PPV) were calculated using equations reported by Carvajal and Rowe (2010)^15^.

### Ethical approval

Ethical approval was obtained from the Human Research Ethics Committee (UPHREC) at the Faculty of Health Sciences, University of Pretoria (Ethics Reference Number: 153/2018) before the study commenced. All protocols and consent forms were executed according to the agreed ethical approval terms and conditions. All clinical samples were obtained from a reference laboratory and not directly from patients, who agreed (through informed consent forms) to our using their specimens for this research. The guidelines stated by the Declaration of Helsinki for involving human participants were followed in the study.

## Results

### MGIT 960 culture

Out of the 205 samples, 63/205 (30.73%) were culture positive, 139/205 (67.80%) were culture negative and three (1.46%) were contaminated. Fifty out of 125 pulmonary were culture positive while 72 were culture negative; three pulmonary specimen were contaminated. Furthermore, 13 extra-pulmonary samples (n = 80) were culture positive while 67 were culture negative (Table 1).

**Table 1:** General characteristics of specimens used in evaluating the smear microscopy, Xpert G4, Xpert Ultra and LPA assays.

Category	Pulmonary samples	Extra-pulmonary samples	Total	Cumulative total
**Specimen type**				
Sputum	117	NA	117	117
abscess	NA^*^	4	4	121
csf	NA	3	3	124
Fluid/ aspirate	NA	18	18	142
swab (superficial)	NA	4	4	146
Tissue	NA	15	15	161
tracheal/aspirate	8	NA	8	169
urine	NA	36	36	205
Total	**125**	**80**	**205**	**205**
**Culture results**				
Culture Contaminated	3	0	3	3
Culture positive	50	13	63	66
Culture negative	72	67	139	205
Total	**125**	**80**	**205**	**205**
**Auramine smear microscopy results**				
Smear (auramine) positive	51	8	59	59
Smear (auramine) negative	74	72	146	205
Total	**125**	**80**	**205**	**205**
**Xpert Ultra results**				
Xpert Ultra positive (TB detected)	76	43	119	119
Xpert Ultra negative (TB undetected)	47	37	84	203
Xpert Ultra Invalid	2	0	2	205
Total	**125**	**80**	**205**	**205**
**Ultra G4 results**				
Xpert G4 positive (TB detected)	49	8	57	57
Xpert G4 negative (TB undetected)	75	71	146	203
Xpert G4 error	1	0	1	204
Xpert G4 invalid	0	1	1	205
Total	**125**	**80**	**205**	**205**
**LPA results**				
LPA positive (TB detected)	107	59	166	166
LPA negative (TB undetected)	18	21	39	205
Total	**125**	**80**	**205**	**205**
**Breakdown of results under culture and smear microscopy categorisation**				
Smear negative (SN)-Culture negative (CN)	65	66	131	131
Smear positive (SP)-Culture positive (CP)	41	7	48	179
SN-CP	9	6	15	194
SP-CN	7	1	8	202
Total	122*	**80**	**202** ^*^	202*

### Auramine smear microscopy

The auramine smear microscopy resulted in 59/205 (28.78%) smear positives and 146/205 (70.73%) smear negatives. In pulmonary specimens, 51/125 (40.80%) were smear positive and 74/125 (59.20%) were smear negative while in extra-pulmonary specimens, 8/80 (10.00%) were smear positive and 72/80 (90.00%) were smear negative.

### GeneXpert MTB/RIF assays

For Xpert Ultra, 76/125 (60.80%) specimens were positive for TB and 47/125 (37.60%) were negative in pulmonary samples, with two (2/125) samples being invalid while 43/80 (53.75%) specimens were positive for TB and 37/80 (46.25%) were negative in extra-pulmonary samples.

For the old XpertG4, 49/125 (39.20%) of specimens were TB-positive and 75/125 (60.00%) were negative, with one (1/125) having invalid results in pulmonary samples while 8/80 (10.00%) specimens were positive for TB and 71/80 (88.75%) were negative, one of which had invalid results in extra-pulmonary specimens.

### GenoType MTBDR*plus* LPA assay

For LPA, 107/125 (85.60%) specimens were TB-positive and 18/125 (14.40%) were TB-negative in pulmonary samples while 59/80 (73.75%) specimens were TB-positive, and 21/80 (26.25%) were TB-negative in extra-pulmonary samples (Table 1).

### Sensitivity, specificity, PPV and NPV

For all the tests, i.e. auramine smear microscopy, Xpert Ultra, Xpert G4 and LPA), the sensitivities were higher in pulmonary samples than extra-pulmonary samples. A similar trend was observed in the specificity of Xpert Ultra, for which pulmonary samples had a higher specificity than extra-pulmonary specimens: 58.57% and 49.25% respectively. However, the specificities of auramine smear microscopy, Xpert G4 and LPA were higher in extra-pulmonary samples than in pulmonary specimens (Table 2).

**Table 2:** Relative diagnostic efficiencies of smear microscopy, Xpert Ultra, Xpert G4 and LPA tests in detecting Mycobacterium tuberculosis from pulmonary and extra-pulmonary samples.

Parameter	Auramine	Xpert Ultra	Xpert G4	LPA
**Pulmonary samples**				
True Positive	41	44	39	40
True Negative	65	41	65	8
False Positive	7	29	7	64
False Negative	9	6	10	10
**Total**	**122**	**120** ^**‡**^	**121** ^**§**^	**122**
Sensitivity (CI), %	82,00 (68,56-91,42)	88,00 (75,69-95,47)	79,59 (65,66-89,76)	80,00 (66,28-89,97)
Specificity (CI), %	90,28 (80,99-96,00)	58,57 (46,17-70,23)	90,28 (80,99-96,00)	11,11 (4,92-20,72)
Positive predictive value (CI), %	85,42 (74,11-92,30)	60,27 (53,00-67,12)	84,78 (73,10-91,95)	38,46 (34,73-42,33)
Negative predictive value (CI), %	87,84 (79,91-92,91)	87,23 (75,87-93,69)	86,67 (78,81-91,91)	44,44 (25,35-65,33)
**Extra-pulmonary samples**				
True Positive	7	9	6	9
True Negative	66	33	65	17
False Positive	1	34	2	50
False Negative	6	4	6	4
Total	**80**	**80**	**79 + 1** ^**^	**80**
Sensitivity (CI), %	53,85 (25,13-80,78)	69,23 (38,57-90,91)	50,00 (21,09-78,91)	69,23 (38,57-90,91)
Specificity (CI), %	98,51 (91,96-99,96)	49,25 (36,82-61,76)	97,01 (89,63-99,64)	25,37 (15,53-37,49)
Positive predictive value (CI), %	87,50 (48,41-98,12)	20,93 (14,66-28,97)	75,00 (40,63-92,93)	15,25 (10,88-20,98)
Negative predictive value (CI), %	91,67 (85,94-95,19)	89,19 (77,89-95,08)	91,55 (86,00-95,03)	80,95 (63,04-91,37)

^†^3 Samples were contaminated and discarded; adding the 3 contaminated isolates equals 205.

^‡^2 Isolates were invalid. Adding them will amount to 122.

^§^1 Isolate was in error, adding it will make up to 122.

^**^1 isolate was invalid. Adding it will amount to 80; # 1 isolate was in error, adding it will make up to 122.

The negative predictive value (NPV) was 100.00% for all tests and samples within this category while auramine smear microscopy had a specificity of 100.00% for all samples within this category. The specificity of Xpert G4 was very high, between 98.46% and 98.48% for both extra-pulmonary and pulmonary samples. The specificity of Xpert Ultra was between 50.00% and 53.49% while that of LPA was below 26% for both pulmonary and extra-pulmonary samples within this category (Table 3).

**Table 3:** Breakdown of the relative diagnostic efficiencies of smear microscopy, Xpert Ultra, Xpert G4 and LPA tests in detecting Mycobacterium tuberculosis from pulmonary and extra-pulmonary samples under different culture and smear results categories.

Parameter	All samples (both pulmonary and extra-pulmonary)				Pulmonary samples				Extra-pulmonary samples			
	Auramine	Xpert Ultra	Xpert G4	LPA	Auramine	Xpert Ultra	Xpert G4	LPA	Auramine	Xpert Ultra	Xpert G4	LPA
	**Smear Negative-Culture Negative (SN-CN)**				**Smear Negative-Culture Negative (SN-CN)**				**Smear Negative-Culture Negative (SN-CN)**			
True Positive	0	0	0	0	0	0	0	0	0	0	0	0
True Negative	131	73	129	25	65	40	64	8	66	33	65	17
False Positive	0	56	2	106	0	23	1	57	0	33	1	49
False Negative	0	0	0	0	0	0	0	0	0	0	0	0
Total	**131**	**129** ^**††**^	**131**	**131**	**65**	**63** ^**††**^	**65**	**65**	**66**	**66**	**66**	**66**
Sensitivity (CI), %	0	0	0	0	0	0	0	0	0	0	0	0
Specificity (CI), %	100,00 (97,22-100,00)	56,59 (47,58-65,29)	98,47 (94,59-99,81)	19,08 (12,75-26,87)	100,00 (94,48-100,00)	63,49 (50,40-75,27)	98,46 (91,72-99,96)	12,31 (5,47-22,82)	100,00 (94,56-100,00)	50,00 (37,43-62,57)	98,48 (91,84-99,96)	25,76 (15,78-38,01)
Positive predictive value (CI), %	0	0	0	0	0	0	0	0	0	0	0	0
Negative predictive value (CI), %	100	100	100	100	100	100	100	100	100	100	100	100
	**Smear Positive-Culture Positive (SP-CP)**				**Smear Positive-Culture Positive (SP-CP)**				**Smear Positive-Culture Positive (SP-CP)**			
True Positive	48	47	43	43	41	40	37	38	7	7	6	5
True Negative	0	0	0	0	0	0	0	0	0	0	0	0
False Positive	0	0	0	0	0	0	0	0	0	0	0	0
False Negative	0	1	3	5	0	1	3	3	0	0	0	2
Total	**48**	**48**	**46** ^**§§**^	**48**	**41**	**41**	**40 + 1** ^***^	**41**	**7**	**7**	**6 + 1** ^**†††**^	**7**
Sensitivity (CI), %	100,00 (92,60-100,00)	97,92 (88,93-99,95)	93,48 (82,10-98,63)	89,58 (77,34-96,53)	100,00 (91,40-100,00)	97,56 (87,14-99,94)	92,50 (79,61-98,43)	92,68 (80,08-98,46)	100,00 (59,04-100,00)	100,00 (59,04-100,00)	100,00 (54,07-100,00)	62,50 (24,49-91,48)
Specificity (CI), %	0	0	0	0	0	0	0	0	0	0	0	0
Positive predictive value (CI), %	100	100	100	100	100	100	100	100	100	100	100	100
Negative predictive value (CI), %	0	0	0	0	0	0	0	0	0	0	0	0
	**Smear Negative-Culture Positive (SN-CP)**				**Smear Negative-Culture Positive (SN-CP)**				**Smear Negative-Culture Positive (SN-CP)**			
True Positive	0	6	2	6	0	4	2	2	0	2	0	4
True Negative	0	0	0	0	0	0	0	0	0	0	0	0
False Positive	0	0	0	0	0	0	0	0	0	0	0	0
False Negative	15	9	13	9	9	5	7	7	6	4	6	2
Total	**15**	**15**	**15**	**15**	**9**	**9**	**9**	**9**	**6**	**6**	**6**	**6**
Sensitivity (CI), %	0,00 (0,00-21,80)	40,00 (16,34-67,71)	13,33 (1,66-40,46)	40,00 (16,34-67,71)	0,00 (0,00-33,63)	44,44 (13,70-78,80)	22,22 (2,81-60,01)	22,22 (2,81-60,01)	0,00 (0,00-45,93)	33,33 (4,33-77,72)	0,00 (0,00-45,93)	66,67 (22,28-95,67)
Specificity (CI), %	0	0	0	0	0	0	0	0	0	0	0	0
Positive predictive value (CI), %	0	100	100	100	0	100	100	100	0	100	0	100
Negative predictive value (CI), %	0	0	0	0	0	0	0	0	0	0	0	0
	**Smear Positive-Culture Negative (SP-CN)**				**Smear Positive-Culture Negative (SP-CN)**				**Smear Positive-Culture Negative (SP-CN)**			
True Positive	0	0	0	0	0	0	0	0	0	0	0	0
True Negative	0	1	1	0	0	1	1	0	0	0	1	0
False Positive	8	7	7	8	7	6	6	7	1	1	1	1
False Negative	0	0	0	0	0	0	0	0	0	0	0	0
Total	**8**	**8**	**8**	**8**	**7**	**7**	**7**	**7**	**1**	**1**	**1**	**1**
Sensitivity (CI), %	0	0	0	0	0	0	0	0	0	0	0	0
Specificity (CI), %	0,00 (0,00-36,94)	12,50 (0,32-52,65)	12,50 (0,32-52,65)	0,00 (0,00-36,94)	0,00 (0,00-40,96)	14,29 (0,36-57,87)	14,29 (0,36-57,87)	0,00 (0,00-40,96)	0,00 (0,00-97,50)	0,00 (0,00-97,50)	0,00 (0,00-97,50)	0,00 (0,00-97,50)
Positive predictive value (CI), %	0	0	0	0	0	0	0	0	0	0	0	0
Negative predictive value (CI), %	0	100	100	0	0	100	100	0	0	0	0	0

^††^2 Isolates were invalid. Adding them will amount to 131.

^‡‡^2 Isolates were invalid. Adding them will amount to 65.

^§§^2 Isolates were invalid/error. Adding them will amount to 46.

^***^1 Isolate was invalid/error. Adding it will amount to 41.

^†††^1 Isolate was invalid/error. Adding that will amount to 7.

In smear positive-culture positive (SP-CP) samples, sensitivity of auramine, Xpert Ultra, Xpert G4 and LPA was between 92–100% while the specificity and NPV were 0.00% for all the assays; PPV was 100% for all assays (Table 3). In smear-negative culture-positive (SN-CP) samples, specificity and NPV were both 0.00% for all samples and tests while PPV for auramine was 0.00% for both pulmonary and extra-pulmonary samples. However, the PPV for auramine Xpert Ultra, Xpert G4 and LPA was 100.00% for all samples, but that of Xpert G4 was 0.00% for extra-pulmonary while that of Xpert Ultra and LPA was 100.00% for extra-pulmonary samples. The sensitivity for auramine was 0.00% for all samples and less than 50.00% for all samples for Xpert Ultra, Xpert G4 and LPA, except for LPA sensitivity in extra-pulmonary samples, which was 66.67% (Table 3).

In smear-positive culture-negative (SP-CN) samples, the sensitivity and PPV and NPV were 0.00% for all four tests in both extra-pulmonary and pulmonary specimens. However, a 100.00% NPV was obtained for Xpert Ultra and Xpert G4 in the combined (both pulmonary and extra-pulmonary) and pulmonary samples (Table 3). Specificity was 0.00% for all four tests for extra-pulmonary samples, 0.00% for auramine and LPA for combined and pulmonary samples. Specificity of Xpert G4 and Ultra were both 12.50% for combined samples, and 14.21% and 14.29% for pulmonary samples respectively (Table 3; Supplementary Dataset).

Rifampicin resistance was detected in 19 isolates, with 16 and one being detected by Xpert Ultra in sputum from SN-CN and SN-CP samples respectively. While the 16 RIF-resistant isolates in SN-CN samples were detected only by Xpert Ultra, the single RIF-resistant isolates in SN-CP samples were detected by both Xpert Ultra and G4. LPA detected two RIF-resistant isolates, which was not confirmed by either Xpert Ultra or Xpert G4, in a single tissue SN-CP sample and in a sputum SP-CP sample (Supplementary Dataset). Other antibiotics resistance conferring-mutations were not determined.

## Discussion

In this study, we compared the performance of the new Xpert Ultra assay with the old Xpert G4 assay, the LPA and auramine smear microscopy for detecting TB in pulmonary and EPTB clinical specimens. All these tests are used as initial rapid diagnostic tests in individuals suspected of having TB^12^. Our results show that the new Xpert Ultra assay has the highest sensitivity (88%) for detecting TB among both pulmonary and EPTB clinical specimens (Table 2), followed by the auramine smear microscopy (82%), LPA (80%) and the old Xpert G4 (79.59%), which had the lowest sensitivity. These results are consistent with the results from recent reports, which showed that Xpert Ultra had a superior sensitivity for TB case detection^10,16^. Chakravorty *et al*. (2017), reported an overall sensitivity of 87.50% for the Xpert Ultra versus a sensitivity of 81.00% for the old Xpert. However, this high sensitivity by the Xpert Ultra led to a lower specificity of 58.57% in pulmonary samples and 49.25% in extra-pulmonary samples as reported in other studies^10,16^. The low specificity could be due to the high rate of Xpert Ultra false positives in mostly pulmonary specimens and the fact that Xpert Ultra has increased limits of detection as compared to other assays^11,16^. Previous TB episodes may also cause false positives due to dead DNA in the sputum^11,16^.

In terms of specificity however, the auramine smear microscopy was higher in both pulmonary and EPTB specimens i.e., 90.28% and 98.51% respectively, than the Xpert G4 (90.28% and 97.01% respectively), Xpert Ultra (58.57% and 49.25% respectively), and the LPA (11.11% and 25.37% respectively), which recorded the lowest specificity among all the tests. It is evident from these results that all the tests are more sensitive to *M. tb* in pulmonary specimens than in EPTB specimens. Except for the Xpert Ultra, all the tests were more specific in detecting the absence of TB in EPTB specimens than in pulmonary clinical samples. The very close sensitivity of the auramine smear microscopy to the Xpert Ultra assay, particularly in pulmonary samples, and its better specificity in detecting the presence and absence of TB in both pulmonary and extra-pulmonary clinical specimens make it a good alternative to these nucleic acid tests. Coupled with its lower costs, lesser skills required and easier application in lower resource settings, our findings support the WHO’s recommendation for the use of the smear microscopy in detecting TB.

Although highly sensitive, the poorer specificity of the Xpert Ultra is a cause for concern as it can lead to high rate of false positives and subsequent placement of such patients on very toxic TB regimens for at least six months. Thus, there is the need to supplement the Xpert Ultra with either culture or the smear microscopy, preferably the latter due to its rapidity and simplicity, to augment for the lower specificity of the Xpert Ultra. Nevertheless, it is also worthy to note that the culture assay, which was used as the gold standard in this study, cannot identify dead, dormant or non-viable *M. tb* strains, a situation that can also lead to false positive results in NAATs that detect DNA in clinical samples. Although MGIT has an analytical sensitivity threshold of 10 bacilli (colony forming units per ml), which is equivalent to Xpert Ultra, the decontamination step reduces the number of bacilli in the sputum. Thus, the MGIT culture is an imperfect gold standard and final diagnosis can be done with other tests (i.e. symptom screen, X-Ray, microscopy). This is a major limitation of the culture tests, making it difficult to provide a true specificity of NAATs when the former is used as a gold standard for evaluating the latter. However, it should be noted that the samples included in this study were from patients who had come for their first TB screening. As a result, there should not be a substantial number of dead *M. tb* DNA in the specimens. Particularly when they had not been given any TB chemotherapy.

In addition, the findings support the use of pulmonary samples for diagnosing TB in suspected patients instead of EPTB ones as the difference in sensitivities for all the four tests between pulmonary and extra-pulmonary samples is substantially wide. It is notable, however, that certain conditions might make it impossible to obtain pulmonary samples for TB diagnosis, specifically from infants and children who cannot produce sputum. And for such cases, the Xpert Ultra and the LPA seem a better diagnostic option due to their higher sensitivity (69.23%) than the other two tests (Table 2). The less than 100% sensitivity of the LPA for smear-positive samples could be due to the presence of novel resistance mechanisms that are undetectable by the LPA probes, inability of culture to detect dead or dormant *M. tb* cells or the processing procedures used^17^.

The specificities and NPVs of all the tests were lowest when culture was positive (SP-CP and SN-CP samples) irrespective of the source of the sample: pulmonary or extra-pumonary. Furthermore, in smear positive-culture positive (SP-CP) samples, the sensitivity was even higher for all the tests, with auramine having the highest sensitivity (100%) followed by Xpert ultra (97.92%), Xpert G4 (93.48%) and LPA (89.58%). Notably, a low sensitivity and PPV were recorded by all the tests in SN-CN and SP-CN samples, albeit with variable specificities that were substantially higher in SN-CN samples (from 19.08% in LPA to 98.47% in Xpert G4) than in SP-CN samples (from 12.50% for both Xpert assays down to 0% for LPA). This shows that a joint absence of TB in both culture and smear microscopy enhances the veracity of the NAATs in predicting the absence of TB than when the two phenotypic tests disagree (in SP-CN samples), which was strongly shown in the Xpert G4 results (Table 3). Contrary to SP-CP samples, where both smear and culture agree and all four tests had higher sensitivies, SN-CP samples, for which smear and culture disagree, reported very low sensitivies for all four tests. These results are consistent with, albeit lower than, the results reported by Dorman *et al*. (2018) where a superior sensitivity of 63% for Ultra was seen vs 44% for the Xpert G4 in SN-CP specimen.

The highest sensitivity of the auramine stain in culture-positive specimens is not surprising as smear positivity normally correlates well with quantitative growth of the TB bacteria^18^. Moreover, because NAATs detect DNA, which are undetectable by culture and auramine, NAATs are more likely to report positive results in specimens for which auramine and culture, which detects cells, are negative. Smear microscopy is still the baseline diagnostic test in resource-limited, high TB-burden countries, specifically on the African continent and South East Asia^12^. However, this is not the case in South Africa as it has rolled out the highest number of Xpert matches than any other country in the world^1^. Smear microscopy is well known for its high specificity, which makes it a reliable test if the specimen does not have the TB bacteria. Our results are therefore consistent with the ones reported in the literature^18,19^.

Rapid diagnosis of EPTB is challenging because of the paucibacillary nature of the disease, causing rare positive smear microscopy results and long incubation time for culture growth^20^. In this study, the Xpert Ultra’s sensitivity was superior to the old Xpert and smear microscopy with pulmonary specimen, albeit the Xpert Ultra had the same sensitivity as the LPA (69.23%) in EPTB specimens. Our results are consistent with results from two studies that have evaluated the performance of Xpert Ultra in EPTB samples^11,21^. This detection improvement in the Xpert Ultra in EPTB samples is due to the larger PCR chamber, the incorporation of two different multicopy targets (IS1081 and IS6110 insertion sequences), and the optimization of PCR and thermal-cycling parameters^16^. The WHO strongly recommends the use of the Xpert assays in EPTB samples such as the cerebrospinal fluid over traditional smear microscopy and culture.

Ninan *et al*. (2016) reported similar sensitivity rates (67%) in smear negative samples for LPA in their study as observed herein with SN-CP specimens, with our LPA also showing a sensitivity of 66.67%^22^. However our LPA sensitivity was a little lower than that reported by Meaza *et al*.^23^. Our Xpert sensitivity in this group of specimens was within the range reported by Perez-Risco *et al*.^11^, with sensitivities ranging from 100% to 0% for different EPTB specimen types. In this study’s EPTB samples, smear microscopy demonstrated the highest specificity (98.51%), followed by the Xpert G4 (97.01%), Xpert Ultra (49.25%) and LPA (25.37%). The assays were all TB-negative in SN-CN extra-pulmonary specimens, which confirms that the patients were negative to TB but presented with other related disease symptoms that could complicate the differential diagnoses.

The Xpert Ultra detected more RIF resistance than the Xpert G4 and the LPA, which supports one of the reasons the Xpert Ultra assay was developed i.e., to overcome the limitations of the old Xpert assays with regards to differentiating certain silent mutations, improving the detection of RIF resistance in mixed infections, and avoiding false-positive results in detecting RIF resistance in paucibacillary specimens^16^.

The use of Primestore MTM (Longhorn Vaccines and Diagnostics, USA) to preserve the specimens used in this study also played a very big role in the high detection of TB in the three molecular assays used in this study. The PrimStore media has been shown to concentrate and enhance *M. tb* detection at low concentrations when using the Xpert assays^24^. The PrimeStore MTM is optimized to inactivate/kill infectious biological pathogens, including *Haemophilus influenzae*, *M. tb*, Gram-Positive/Negative bacteria, and viruses (Daum *et al*., 2015). It disrupts/lyses lipid membranes and inactivates nucleases^14^. Moreover, PrimeStore MTM preserves and stabilizes RNA and DNA at ambient temperature and is compatible with manual and high throughput RNA/DNA isolation kits^14^.

Major limitations to this study includes the relatively smaller sample size, the absence of samples from totally healthy patients to serve as negative controls, the use of samples from a single reference laboratory (although this laboratory serves about 200 clinics and at least six academic hospitals in three provinces in South Africa), and the inability of the MGIT to identify dormant or dead cells. In addition, as primestore MTM was used to concentrate our DNA from all samples, we suspect that it could have given an advantage to the molecular assays in terms of sensitivity as low bacilli load in specimens can be killed during decontamination step in culture assays. Notably, the sensitivity and specificity values of the NAATs might be impacted by the MGIT, which was used as a gold standard; specifically, when positive samples are identified as negative by the culture (MGIT) due to low bacillary load. This could explain the lower specificity of the LPA and Xpert Ultra.

## Conclusion

A significant diagnostic performance of the GeneXpert Ultra assay in the detection of TB and RIF resistance was observed, confirming its efficiency for rapid diagnosis of TB in either pulmonary or extra-pulmonary specimens. Nevertheless, caution is advised in interpreting or using the Xpert Ultra’s specificity (TB-negative predictions) results clinically without a confirmation with culture or smear microscopy. The LPA showed relatively comparable diagnostic accuracy in detecting TB in all clinical specimens, albeit it was poorly specific than the Xpert Ultra; more studies are needed to confirm these findings on the two molecular assays using a better gold standard.

## Supplementary information

Supplementary dataset

## References

[1] WHO. *Global Tuberculosis Report 2017*. *Who*, doi:WHO/HTM/TB/2017.23 (2017).

[2] Osei Sekyere, J., Reta, M. A., Maningi, N. E. & Fourie, P. B. Antibiotic Resistance of Mycobacterium tuberculosis complex in Africa : A Systematic Review of Current Reports of Molecular Epidemiology, Mechanisms and Diagnostics. *J. Infect.*, 0 10.1016/j.jinf.2019.10.006, https://linkinghub.elsevier.com/retrieve/pii/S0163445319303160 (2019). 10.1016/j.jinf.2019.10.006 31629017

[3] Churchyard, G. J. *et al*. Tuberculosis control in South Africa: Successes, challenges and recommendations. *South African Med. J*., 10.7196/SAMJ.7689 (2014). 10.7196/samj.7689 24893501

[4] Helb D (2010). Rapid detection of Mycobacterium tuberculosis and rifampin resistance by use of on-demand, near-patient technology. J. Clin. Microbiol..

[5] Theron, G. *et al*. Determinants of PCR performance (Xpert MTB/RIF), including bacterial load and inhibition, for TB diagnosis using specimens from different body compartments. *Sci. Rep*. **4** (2014). 10.1038/srep05658 PMC5375978 25014250

[6] Denkinger CM (2014). Xpert MTB/RIF assay for the diagnosis of extrapulmonary tuberculosis: A systematic review and meta-analysis. Eur. Respir. J..

[7] Zeka AN, Tasbakan S, Cavusoglu C (2011). Evaluation of the GeneXpert MTB/RIF assay for rapid diagnosis of tuberculosis and detection of rifampin resistance in pulmonary and extrapulmonary specimens. J. Clin. Microbiol..

[8] Köser CU, Feuerriegel S, Summers DK, Archer JAC, Niemann S (2012). Importance of the genetic diversity within the Mycobacterium tuberculosis complex for the development of novel antibiotics and diagnostic tests of drug resistance. Antimicrobial Agents and Chemotherapy.

[9] Chakravorty S (2012). Rapid, high-throughput detection of rifampin resistance and heteroresistance in Mycobacterium tuberculosis by use of sloppy molecular beacon melting temperature coding. J. Clin. Microbiol..

[10] Dorman, S. E. *et al*. Xpert MTB/RIF Ultra for detection of Mycobacterium tuberculosis and rifampicin resistance: a prospective multicentre diagnostic accuracy study. www.thelancet.com/infection *Lancet Infect Dis* **18**, 76–84 (2018). 10.1016/S1473-3099(17)30691-6 PMC6168783 29198911

[11] Perez-Risco, D., Rodriguez-Temporal, D., Valledor-Sanchez, I. & Alcaidea, F. Evaluation of the Xpert MTB/RIF Ultra Assay for Direct Detection of Mycobacterium tuberculosis Complex in Smear-Negative Extrapulmonary Samples. *J. Clin. Microbiol*. **56** (2018). 10.1128/JCM.00659-18 PMC6113499 29950333

[12] WHO. *World Health Organization Model List of Essential In Vitro Diagnostics*. (2018).

[13] Maningi, N. E. *et al*. Multi- and Extensively Drug Resistant Mycobacterium tuberculosis in South Africa: a Molecular Analysis of Historical Isolates. *J. Clin. Microbiol*. **56**, JCM.01214-17 (2018). 10.1128/JCM.01214-17 PMC5925707 29514936

[14] Daum LT (2014). A molecular transport medium for collection, inactivation, transport, and detection of Mycobacterium tuberculosis. Int. J. Tuberc. Lung Dis..

[15] Carvajal DN, Rowe PC (2010). Research and Statistics: Sensitivity, Specificity, Predictive Values, and Likelihood Ratios. Pediatr. Rev..

[16] Chakravorty, S. *et al*. The new Xpert MTB/RIF ultra: Improving detection of Mycobacterium tuberculosis and resistance to Rifampin in an assay suitable for point-of-care testing. *MBio* **8** (2017). 10.1128/mBio.00812-17 PMC5574709 28851844

[17] Nathavitharana, R. R. *et al*. Accuracy of line probe assays for the diagnosis of pulmonary and multidrug-resistant tuberculosis: a systematic review and meta-analysis. *Eur. Respir. J*. **49** (2017). 10.1183/13993003.01075-2016 PMC5898952 28100546

[18] Hooja S (2011). Comparison of Ziehl Neelsen & Auramine O staining methods on direct and concentrated smears in clinical specimens. Indian J. Tuberc..

[19] Agrawal M, Bajaj A, Bhatia V, Dutt S (2016). Comparative study of GeneXpert with ZN stain and culture in samples of suspected pulmonary tuberculosis. J. Clin. Diagnostic Res..

[20] Rahman, F. *et al*. Comparison of Different Microscopic Methods with Conventional TB Culture. *Stamford J. Microbiol*., 10.3329/sjm.v1i1.9133 (1970).

[21] Bahr, N. C. *et al*. Diagnostic accuracy of Xpert MTB/RIF Ultra for tuberculous meningitis in HIV-infected adults: a prospective cohort study. *Lancet Infect. Dis*., 10.1016/S1473-3099(17)30474-7 (2018). 10.1016/S1473-3099(17)30474-7 PMC5739874 28919338

[22] Ninan MM, Gowri M, Christopher DJ, Rupali P, Michael JS (2016). The diagnostic utility of line probe assays for multidrug-resistant tuberculosis. Pathog. Glob. Health.

[23] Meaza, A. *et al*. Evaluation of genotype MTBDRplus VER 2.0 line probe assay for the detection of MDR-TB in smear positive and negative sputum samples. *BMC Infect. Dis*. **17** (2017). 10.1186/s12879-017-2389-6 PMC5392973 28415989

[24] Daum LT (2016). Xpert® MTB/RIF detection of Mycobacterium tuberculosis from sputum collected in molecular transport medium. Int. J. Tuberc. Lung Dis..

